# A Video Demonstration of Preserved Piloting by Scent Tracking but Impaired Dead Reckoning After Fimbria-Fornix Lesions in the Rat

**DOI:** 10.3791/1193

**Published:** 2009-04-24

**Authors:** Ian Q. Whishaw, Boguslaw P. Gorny

**Affiliations:** Department of Neuroscience, Canadian Centre for Behavioural Neuroscience, University of Lethbridge

## Abstract

Piloting and dead reckoning navigation strategies use very different cue constellations and computational processes (Darwin, 1873; Barlow, 1964; O’Keefe and Nadel, 1978; Mittelstaedt and Mittelstaedt, 1980; Landeau et al., 1984; Etienne, 1987; Gallistel, 1990; Maurer and Séguinot, 1995). Piloting requires the use of the relationships between relatively stable external (visual, olfactory, auditory) cues, whereas dead reckoning requires the integration of cues generated by self-movement. Animals obtain self-movement information from vestibular receptors, and possibly muscle and joint receptors, and efference copy of commands that generate movement. An animal may also use the flows of visual, auditory, and olfactory stimuli caused by its movements. Using a piloting strategy an animal can use geometrical calculations to determine directions and distances to places in its environment, whereas using an dead reckoning strategy it can integrate cues generated by its previous movements to return to a just left location. Dead reckoning is colloquially called "sense of direction" and "sense of distance."

Although there is considerable evidence that the hippocampus is involved in piloting (O’Keefe and Nadel, 1978; O’Keefe and Speakman, 1987), there is also evidence from behavioral (Whishaw et al., 1997; Whishaw and Maaswinkel, 1998; Maaswinkel and Whishaw, 1999), modeling (Samsonovich and McNaughton, 1997), and electrophysiological (O’Mare et al., 1994; Sharp et al., 1995; Taube and Burton, 1995; Blair and Sharp, 1996; McNaughton et al., 1996; Wiener, 1996; Golob and Taube, 1997) studies that the hippocampal formation is involved in dead reckoning. The relative contribution of the hippocampus to the two forms of navigation is still uncertain, however.  Ordinarily, it is difficult to be certain that an animal is using a piloting versus a dead reckoning strategy because animals are very flexible in their use of strategies and cues (Etienne et al., 1996; Dudchenko et al., 1997; Martin et al., 1997; Maaswinkel and Whishaw, 1999). The objective of the present video demonstrations was to solve the problem of cue specification in order to examine the relative contribution of the hippocampus in the use of these strategies.  The rats were trained in a new task in which they followed linear or polygon scented trails to obtain a large food pellet hidden on an open field. Because rats have a proclivity to carry the food back to the refuge, accuracy and the cues used to return to the home base were dependent variables (Whishaw and Tomie, 1997). To force an animal to use a a dead reckoning strategy to reach its refuge with the food, the rats were tested when blindfolded or under infrared light, a spectral wavelength in which they cannot see, and in some experiments the scent trail was additionally removed once an animal reached the food. To examine the relative contribution of the hippocampus, fimbria–fornix (FF) lesions, which disrupt information flow in the hippocampal formation (Bland, 1986), impair memory (Gaffan and Gaffan, 1991), and produce spatial deficits (Whishaw and Jarrard, 1995), were used.

**Figure:**
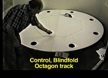


## Protocol

### Animals

Twelve adult female Long–Evans rats (University of Lethbridge vivarium), weighing 250-300 gm, were housed in groups in wire mesh cages in a laboratory with room temperature maintained at 20–21°C and lighted on a 12 hr light/dark cycle (8 A.M. to 8 P.M.). Six rats received sham operations and six received fimbria–fornix lesions before testing.

### Surgery

For sterile surgery, the rats were anesthetized with sodium pentobarbital (40 mg/kg, i.p.) and atropine methyl nitrate (5 mg/kg, i.p.). To make fimbria–fornix lesions, 1.5 mA cathodal current was passed for 40 sec through 00 stainless steel insect pins, insulated with epoxylite except at the surface of their tips. Lesions were made at two sites in each hemisphere using coordinates in reference to bregma and the surface of the dura: 1.3 mm posterior, 1.5 mm lateral, and 3.6 mm ventral, and 1.5 mm posterior, 0.5 mm lateral, and 3.3 mm ventral (Whishaw and Jarrard, 1996). The control rats received anesthesia only, and for 48 h postoperatively the rats were medicated for the control of pain.

All animal use was in accordance with protocols approved by the University of Lethbridge Animal Care and Use Committee.

### Feeding

Feeding was restricted to maintain the rats at 90% of their expected body weights. Large (750 mg) rodent pellets (Bio-Serv, Frenchtown, NJ) were used for reward during behavioral testing. Rats reliably carry these pellets to a refuge for eating (Whishaw et al., 1995a,b). After testing each day, the rats were supplementally fed with LabDiet Laboratory Rodent Pellets in their home cage.

### Apparatus

The open field consisted of a 204-cm-diameter circular wooden table, similar to a Barnes’s spatial testing apparatus (Barnes, 1979), that was painted white and elevated 64 cm above the floor (Whishaw and Maaswinkel, 1998). Eight 11.5-cm-diameter holes were cut in the table, spaced equidistant around its perimeter and centered 13.5 cm from the table’s edge (Fig. 1*A*). A cage, similar to a rat’s home cage, could be inserted beneath a hole to serve as a refuge. The apparatus was located in a test room in which many cues, including windows covered by blinds, counters, a refrigerator, cupboards, a desk with computers, etc., were present. A camera was located above the center of the table so that the behavior of the animals could be videorecorded (Whishaw and Tomie, 1997).

### Strings and scent

The rats were trained to follow a scented string to the food pellet. The string was ;2 mm diameter, but pieces were of variable length and were placed on the table to form varying patterns on the table (Fig. 1*A,B*). The strings were scented with almond extract. The odor remained on the string for  20 min after it was wiped onto the string with a soaked piece of cotton gauze. Preliminary experiments indicated that rats trained to follow the scented string (Fig. 1*C*) did not follow an odor tract left by a piece of string that had just been removed.

### Masks and blindfolds

Masks and blindfolds, used to control the rats use of visual cues, were constructed of felt and attached by a Velcro collar fixed around a rat’s ( Fig. 2 ). They were fastened across a rat’s face by an elastic chin strap that was attached to the neck collar. The elastic strap was flexible so that a rat could grasp food pellets with its mouth and chew and swallow them. A mask allowed the rats to see, whereas a blindfold occluded vision. The effectiveness of the blindfolds was tested on rats trained to swim to a visible platform located in a swimming pool. Well trained rats wearing a mask swam directly to the platform from any starting point on the periphery of the pool, whereas rats wearing the blindfold swam around the edge of the pool or swam in a haphazard manner. The rats were adapted to masks and blindfolds by having them wear the apparel for at least 30 min/day for 5 days before testing. Before the formal tests, a mask or blindfold was placed on the animals for 30 min before the test.

Infrared testing In addition to testing the rats while their vision was occluded by blindfolds, some of the experiments were performed under infrared light. The test room was light proof, and the room lights were turned off. Under infrared light, the animals’ movements were recorded with a Sony infrared camera. The experimenter used an infrared spotter to orient in the test room.

### Training

All of the training was conducted with straight scented trails. A piece of string of variable length, scented with almond extract, was stretched from the edge of the refuge hole and led to the food pellet (Fig. 1). Initially, the string was quite short (,10 cm) and led in different directions, but as the animals became proficient in following the string, its length was increased to up to .150 cm. Training lasted 14 days, by which time the rats were proficient in following the string to retrieve food under both mask and blindfolded conditions.

### Analysis

The behavior of the rats was filmed on all of the tests. From the ongoing trials and the video recordings the following behavioral measures were made.

**Correct trial.** A correct trial was a trial during which a rat found a food pellet and returned directly to the starting hole without stopping at any other potential exit hole.

**Retrieval.** A retrieval was defined as an exit from the home cage and a return with a food pellet.

**Error.** An incorrect trial was one during which a rat found a food pellet but stopped at one of the other potential exits before returning to the exit from which its excursion began. A rat was deemed to have stopped at an exit if its snout was brought to within  2 cm of a hole (errors were usually unambiguous because the rats stopped and inserted their heads into the holes). In some experiments, if the rat followed the string home, rather than taking a more direct route, that was classified as a error.

**Travel distance.** The outward and homeward distances were analyzed with a movement-tracking device to compute distance traveled.

**Response times.** Using a stop watch, an observer recorded separately the time taken to find a food pellet and the time taken to return to the home cage with the food.

**Heading angles.** Measures of heading angles were made after a rat retrieved a food pellet and began its homeward trip. Once the rat had moved one body length with the food, the angle that it was pointing, measured through the long axis of the animal’s body relative to the most direct line to the refuge, was measured.

**Statistical analysis**

Group comparisons were made using ANOVA’s and *t* tests (Winer, 1962).

### Histology

At the completion of the experiments, the rats were deeply anesthetized and perfused with saline and saline–formalin, and the brains were removed and stored in a 30% sucrose–formalin solution. The brains were cut in 40 µm sections on a cryostat, and alternate sections were stained with cresyl violet and for acetylcholinesterase.

### Procedure

Testing began once the rats were reliably following the string to retrieve the food. The following four tests were administered. Return accuracy on a linear scent track . A scented string 125 cm long and an unscented string of the same length were both stretched in a straight line on the table. The location of the strings varied from trial to trial, and trials were given in a pseudo-random order, with one trial given each day. Each rat received three trials while wearing a mask and three trials while wearing a blindfold. Return accuracy on a linear track with scent removed. The rats were tested with strings of length 20, 30, 50, 100, and 150 cm. Strings were placed on the table so that one end was at the target distance and the other led into the rats’ refuge and then to the experimenter. Once a rat reached the food pellet, the string was gently pulled from the table. All of the rats received three trials wearing masks and three trials wearing blindfolds at each of the distances. Order of distance presentation varied from trial to trial as did string direction. Return accuracy from a polygon scent track . The rats received four tests with the scented track positioned on the table in a polygon pattern (curved or angular). The length, direction, and pattern of the string arrangement were different for each test, but in all cases the distance home from the food location was much shorter than the string distance.  For three tests, the rats performed once with masks and once with blindfolds. For the fourth test, the rats wore no head gear, and one trial was given in normal light and one trial was given in infrared light. Travel distance on an octagonal scent track . The string led from the refuge cage and then formed an octagon (75 cm diameter) in the center of the table. No food pellet was present. Each rat received one trial under room lights on 1 day and another trial under infrared light on a second day.  Once a rat reached the octagon, the string connecting the refuge with the octagon was removed. If a rat left the octagon and entered the refuge the trial was complete. If a rat completed four complete rotations around the octagon, the trial was ended by removing the rat.  Test from a novel location, with and without vision. Each rat completed two trials following the scented string from a novel location. On one trial the rats wore a mask, and on the second trial they wore blindfolds.

### General behavioral observations

After a rat in a refuge cage was placed beneath a hole, the rat typically poked its head out of the hole a number of times before it exited. It exited by pulling itself up with its forepaws and pushing with its hind paws. Once on the table, it paused and scanned the table in search of the scented string. On its outward journeys it walked along the string, which it sniffed with lateral scanning motions of the head. If a rat deviated away from the string, it quickly circled to relocate the string. Once a rat found a piece of food, it grasped it in its mouth and set off for the refuge. Once it arrived at the refuge, it inserted its head into the hole and adjusted the position of its feet so that it could drop down into the cage beneath the hole. In general, travel speed home was faster than travel speed out. If the rat was wearing a mask its travel speed was slightly slower than when it was sighted, both when tracking and returning to the refuge. Travel speed under infrared light was typically faster than travel speed under masked conditions. In all of the experiments, the major group differences were caused by the very poor performance of the fimbria–fornix rats when they could not use vision or olfaction. Although the fimbria–fornix rats were able to return home quickly in the light and to follow the string to the refuge in the absence of vision, they became lost and frequently ate their food on the table in the absence of these cues.

### Return accuracy on a linear scent track

To evaluate tracking accuracy, a straight scented string and a straight nonscented string were placed on the table. On all trials, when masked and blindfolded, both the control and the fimbria– fornix rats followed the scented string to the food and ignored the nonscented string. They also returned directly back to the refuge, along the path of the scented string. Figure 3 illustrates the return paths of the control and fimbria–fornix rats on one tracking problem. Although there were no group differences, there were significant differences in response times. The rats were faster in the mask than in the blindfolded condition (*F*(1,10) = 10.4; *p* <0.01), and they were faster in returning to the home cage with the food than they were in traveling out to the food (*F*(1,10) = 2.7;  *p* < 0.05).

### Return accuracy on a linear track with scent removed

The rats were presented with strings of different length (Fig. 4*A*), and the strings were removed just as the rats grasped the food, thus requiring that the rats return home without the scented track, either with vision or when blindfolded. The main findings were that the control rats were equally accurate in sighted and blindfolded conditions. When sighted, the fimbria–fornix rats performed almost as well as the control rats; when blindfolded, they made more errors, had longer return times, and had more deviant heading directions than they did when sighted and than did the control rats. Errors A measure of errors, visits to incorrect holes on the return trip, gave significant effects of group (*F*(1,10) = 26.1; *p* = 0.005), visual condition (F(1,10) = 23.8; p = 0.006), and visual condition by group (*F*(1,10) = 23.8; *p* = 0.006). All of these effects were attributable to errors made by the fimbria–fornix rats (Fig. 4B). There were also significant effects of distance traveled out versus back (*F*(4,40) = 3.81; *p* = 0.01) and distance by group (*F*(4,40) = 3.8; *p* = 0.01). The interaction effect was attributable mainly to the much poorer performance of the fimbria–fornix group in the blindfolded condition than in the sighted condition. The fimbria– fornix rats did make errors in the light, but these errors occurred mainly when the strings were adjacent to other holes, rather than when they occupied the center of the board. Sighted fimbria– fornix rats had a strong tendency to stop at incorrect holes if they were adjacent to their route home.

### Time

A measure of time taken to return to the refuge with the food gave a significant effect of group (*F*(1,10) = 15.171; *p* = 0.003), visual condition (*F*(1,10) = 15.5; *p* = 0.028), and visual condition by group (*F*(1,10) = 15.1; *p* = 0.003). All of the significant effects of time were attributable to the slow returns of the fimbria–fornix rats when they were blindfolded (Fig. 4*C*). On many trials they lost their way, ate their food, and only reached the refuge after haphazard walks around the foraging table. Although the fimbria–fornix rats returned more quickly at the shortest distance, the effect of distance did not quite reach significance (*F*(4,40) = 2.49; *p* = 0.058).

### Heading angle

Heading angles, consisting of the direction the rat pointed after traveling one body length with the food relative to the straight line direction to the food, are summarized by group, condition, and distance in Figure 5. There were significant effects of group (*F*(1,10) = 14.9; *p* = 0.003), visual condition (*F*(1,10) = 13.8; *p* = 0.004), and visual condition by group (*F*(1,10) = 13.1; *p* = 0.05). These effects were caused by the more deviant angles of the rats with FF lesions. There were also significant effects of distance traveled to reach the food (*F*(4,40) = 4.44; *p* = 0.04) and distance traveled by group (*F*(4,40) = 2.94; *p* = 0.03). These effects were attributable to the more deviant heading angles of the blindfolded fimbria–fornix rats at longer distances. Behavioral analysis When the way in which the rats retrieved the food and turned toward home was examined, differences were observed between the turning movements made by control and fimbria–fornix rats (Fig. 6). The control rats typically stretched forward to retrieve the food and then recoiled along the main axis of their body so that they faced in the direction from which they had come. Forward stretching was much less obvious in the fimbria–fornix rats, which did not recoil and turn but simply pivoted as they walked. In blindfolded conditions, the first response of the fimbria–fornix rats with the food was often to begin to eat before attempting to return home. Counts on whether the rats recoiled along their outward path or moved in some other direction gave a significant group effect (*F*(1,10) = 39.2; *p* < 0.001).

### Return accuracy from polygon scent tracks

The rats were tested on three polygons under masked and blindfolded conditions (Fig. 7*A*). Response times and distances out and back were averaged for the three problems. The main findings were that the control rats took the most direct route home under both masked and blindfolded conditions, whereas the fimbria–fornix rats took the direct route home only while they were sighted. An examination of the behavior of the rats showed that both control and fimbria–fornix rats tracked the string on the outward trip in both mask and blindfolded conditions. The control rats always returned directly to the starting hole via the shortest route under both visual and blindfolded conditions. The fimbria–fornix group returned directly home when sighted, but when blindfolded they followed the scent track home. Accordingly, the outward distance for both groups of rats in both conditions was very similar to the outward string length. The homeward distance for the control and sighted fimbria–fornix rats was very close to the shortest distance back to the starting hole. The travel distance of the blindfolded fimbria–fornix rats was much longer than the string distance back. This was because many of the rats walked off course and had to find their way back to the string, or else they circled, as if searching for the refuge, before they reached it.

### Masks or blindfolds

Travel times Although there was no group effect for time (*F*(1,20) = 3.39; *p* = 0.09, the effects of visual condition (*F*(1,10) = 40; *p* = 0.001) and direction traveled (*F*(1,20) = 5.0; *p* = 0.49) were significant. The fimbria–fornix rats were faster on their outward trips than were the control rats, especially in the mask condition, and there were no effects of blindfolding within the groups. What is perhaps more important, the interactions between group and visual condition (*F*(1,10) = 20.5; *p* = 0.001) and group by direction traveled (*F*(1,10) = 10.8; *p* = 0.008) were significant. The interactions were attributable to slower returns in both control and fimbria–fornix rats in the blindfold condition and the especially slow return times for the fimbria–fornix rats in the blindfolded condition (Fig. 7*B*).

### Travel distance

Analysis of the travel distance gave significant effects for all of the main effects, groups (*F*(1,20) = 13.8; *p* = 0.004), visual condition (*F*(1,10) = 24; *p* = 0.001), and direction (*F*(1,20) = 16.3; *p* < 0.002). Outward travel distances in both groups in both visual conditions were similar and approximated the string distance, but there were group differences in homeward distance, as indicated by the significant interactions between group by visual condition (*F*(1,10) = 14.92; *p* = 0.003), group by direction (*F*(1,10) = 7.52; *p* = 0.001), and vision by direction (*F*(1,10) = 5.9; *p* = 0.03). There was no effect of blindfolding on the homeward trips of the control group, whose return trip approximated the distance of the shortest route home, but the fimbria–fornix group took long routes when blindfolded (Fig. 7*C*).

### Room or infrared light

The rats were given one polygon problem under room light and another under infrared light, and their return paths are illustrated in Figure 8. All six control rats returned directly to the home under light and dark conditions, whereas five of the fimbria–fornix rats returned directly home under light conditions. All of the fimbria–fornix rats returned home along the track in the dark (Fig. 8*A*). As occurred in the blindfold conditions, return latencies for the fimbria–fornix rats in the dark were significantly higher than latencies for the fimbria–fornix rats in the light or for the control group in either the light or dark  ( *p* < 0.01) (Fig. 8*B*).

### Travel distance on an octagonal track

The rats were allowed access to an octagonal tract via a string bridge. Once they were on the octagonal track, the connecting bridge to their home was removed (Fig. 9*A*). The control rats each made a single trip around the track, and when they arrived back at the starting location without finding food, they went to the refuge hole and entered the refuge cage. The fimbria–fornix rats made many trips around the octagon (Fig. 9*B*), and most were removed at the end of their fourth circuitry. Thus, the control rats made fewer circles around the octagon (control mean = 1 vs FF mean = 3.83) and spent much less time tracking the string (*F*(1,10) = 15.3; *p* = 0.003) than did the fimbria–fornix rats (Fig. 9*C*).

### Test from a novel location, with and without vision

The return routes and choices made by control and fimbria–fornix rats in tests in which their refuge cage was moved to a new location are shown in Figure 10. When masked, all of the rats, with the exception of two control rats, returned to the familiar home location (Fig. 10*A*). The two control rats returned directly to the new home location. When blindfolded, all of the control rats returned by fairly direct paths to the new location. The fimbria–fornix rats were inaccurate and encountered incorrect locations or returned to the new location by following the string (Fig. 10*B*).

### Figures:


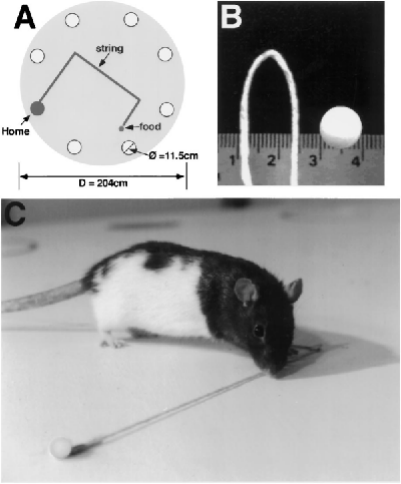


**Figure 1. A**, The foraging table   showing the location of the refuge hole (F) and a string in one of its   configurations with a food pellet located at its end. **B**,   An example of the string that the rats tracked and food pellets that they   carried back to the refuge. **C**, An example of a rat   tracking along the string to the food pellet.


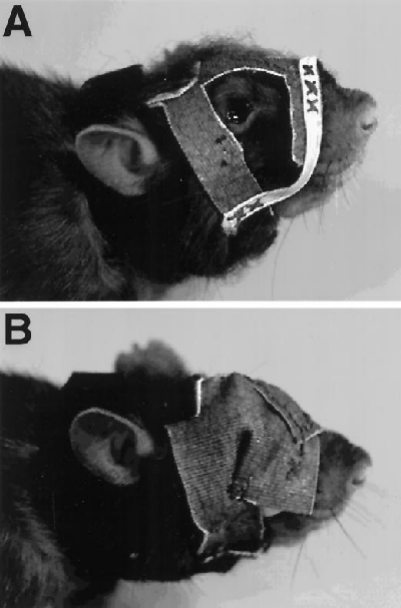


**Figure 2.** Masks (*A*) and   blindfolds (*B*). Both kinds of headwear are fixed to a rats head by   Velcro collars. An elastic chin strap holds the headwear against a rat’s   face but still allows the rat to open its mouth and chew.


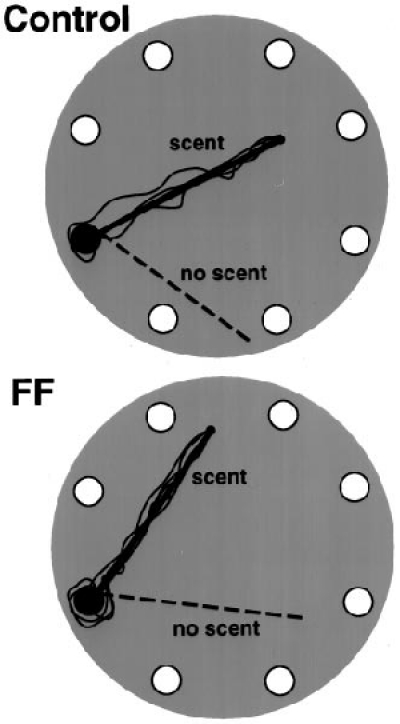


**Figure 3.** Illustrations of the   homeward trajectories (*solid lines*) of the control (*top*)   and fimbria–fornix (*FF*) rats along a scented string in the   blindfold condition. The *dotted line* is a nonscented string.


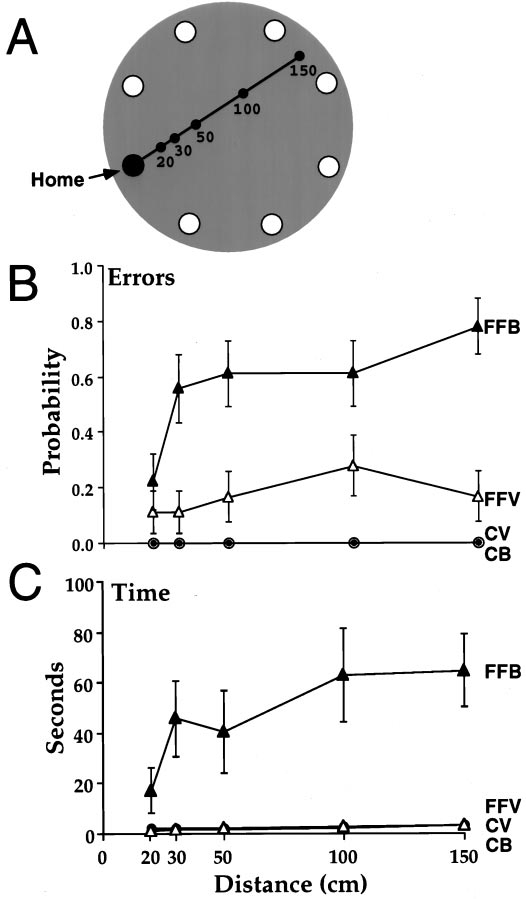


**Figure 4. A,** The rats traveled one   of five linear distances to find a food pellet. The scent string was   removed when they reached the food pellet. **B**, Errors as a   function of distance on the return trip (mean and SE) consisted of a visit   to an incorrect hole. **C**, Latencies to return home as a   function of distance (mean and SE). *FFB*, Fimbria–fornix blindfold;   *FFV*, fimbria–fornix vision; *CV*, control vision;   *CB*, control blindfold.


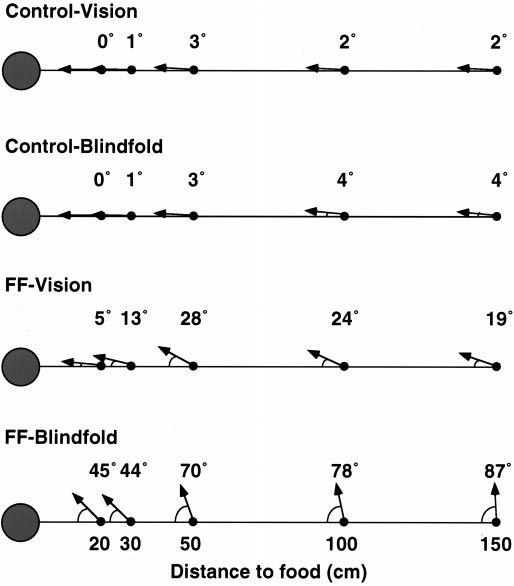


**Figure 5.** Turning angles of rats   after food retrieval as a function of distance traveled to reach the food.   Note that the fimbria–fornix rats were inaccurate at all distances when   blindfolded.


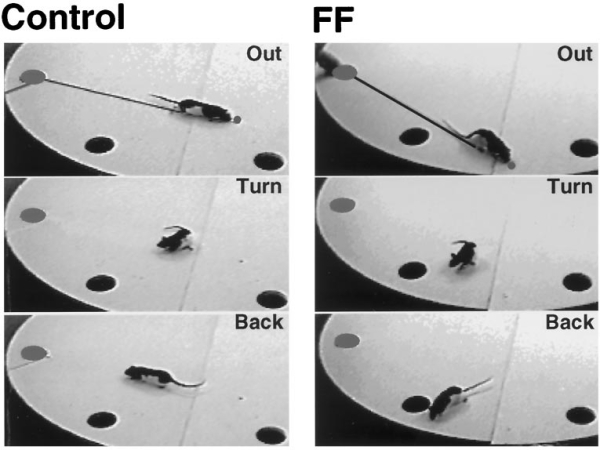


**Figure 6.** Turning strategies of   control and fimbria–fornix rats on retrieving a food pellet when the scent   string was removed. The control rat stretches forward to retrieve the food   and then recoils backward and pivots to face the refuge. The fimbria–fornix   rat walks over to the food and fails to pivot back to the return path.


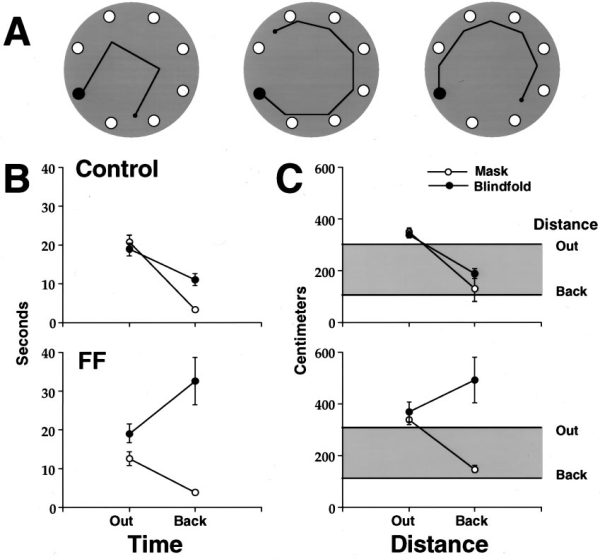


**Figure 7. A,** Three polygon string   tracks that the rats were required to follow. **B**, Time   (mean and SEs on the three problems) taken to travel to the food   (*Out*) and return (*Back*) in control and fimbria–fornix   (*FF*) rats in mask and blindfold conditions. **C**,   Distance traveled to reach the food by control and fimbria–fornix rats in   masked and blindfolded conditions. The *top portion* of the *gray   bars* (*Out*) represents the distance to the food along the   string. The *bottom portion* of the *gray bars* (*Back*) represents the shortest distance from the food to the home   refuge. Note that the performance of the groups is quite similar and   approximates the string out and shortest back distance, with the exceptions   of the return back performance of the fimbria–fornix rats when   blindfolded.


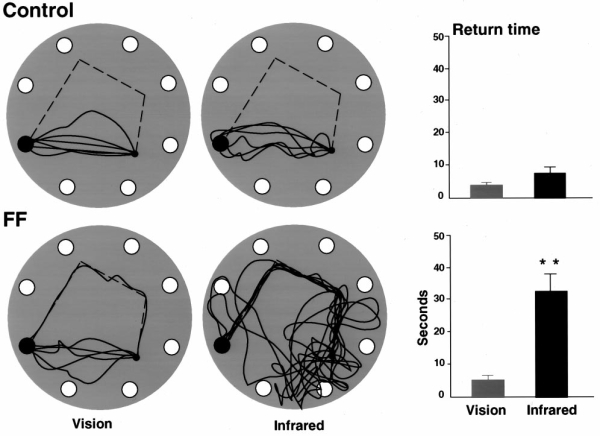


**Figure 8. A**, Homeward trajectories   for control and fimbria–fornix (*FF*) rats under room light and   infrared light on one polygon string problem. Returns of fimbria–fornix   rats were inaccurate in the dark, because they were largely following the   scent string back to the refuge. Return latencies were mean and SEs. Note   the significantly longer latencies of the fimbria–fornix rats under   infrared light.


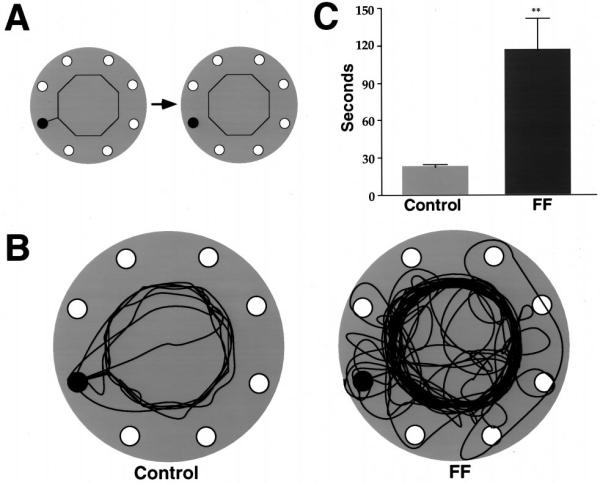


**Figure 9. A**, A string led from the   refuge hole to an octagonal circuit on which there was no food. Once the   rat was on the octagon, the string connecting it to the home cage was   removed. **B**, Travel paths made by control and   fimbria–fornix rats. Note that the control rats averaged one circle on the   octagon before returning to the home cage, whereas the fimbria–fornix rats   made many turns around the octagon. **C**, Time (mean and SE)   spent traveling on the octagon for control and fimbria–fornix rats.


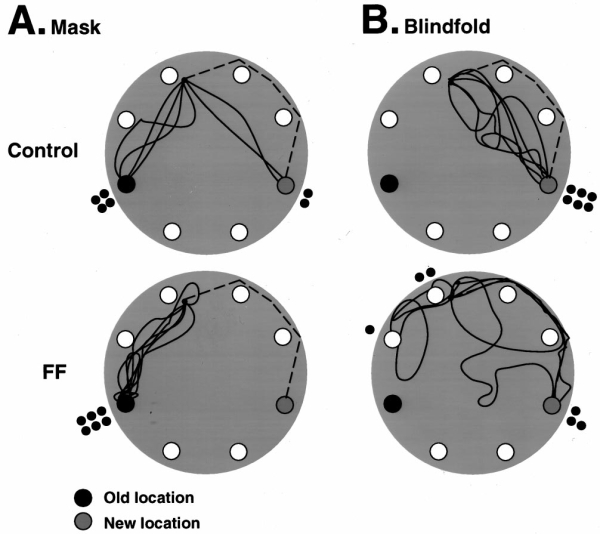


**Figure 10.** Paths and destinations   of control and fimbria–fornix rats when the home cage was moved to a new   location (*shaded refuge*). Note that in the mask condition in which   the rats could see, most control rats and all fimbria–fornix rats carried   the food to the old home location. In the blindfold condition in which the   rats could not see, all control rats carried food to the new home location   via a direct route, whereas fimbria–fornix rats reached other nearby holes   or followed the string back to the new home location.

## Discussion

The experiments examined the contribution of the hippocampus to piloting versus dead reckoning navigation by exploiting a novel task in which rats were challanged to return home after an outward trip following a scented string from their refuge to a food pellet located at the end of the string. Control rats navigated efficiently using both spatial and dead reckoning strategies. The rats with fimbria–fornix lesions successfully navigated using a spatial strategy but were impaired when forced to use dead reckoning. These results are consistent with the hypothesis that the integrity of the hippocampal formation is essential for dead reckoning. This may also be the first demonstration in tracking in an animal other than the dog.

### Task demands

The novel feature of this study was the use of a scented string that allowed the subjects to track an odor trail to a food pellet. The use of tracking facilitates an analysis of the rats’ homing behavior in a number of ways. First, the rats’ outward path and the cue are specified. Rats are very capable of using surface olfactory cues (Maaswinkel and Whishaw, 1999), and so the use of a scented string forces them to focus on a particular cue. Second, all of the rats could be presented with exactly the same outward journey. This removes the variability that occurs when they take haphazard and individual outward routes in search of randomly located food. The third an important feature of the procedure is that it was possible to determine when the rats switched cues and navigation strategies between the outward and homeward trip. In the course of the study, the rats received a large number of tracking problems, which included linear tracks, polygon tracks, and an octagonal track. What was similar in all of the problems, however, was the comparison that was made between the rats’ ability to navigate home when they could use spatial cues (vision or olfaction) and their ability to return home using dead reckoning cues (when blindfolded or tested in infrared light).

### Cue use

When they were sighted, both the control and the fimbria–fornix rats traveled relatively directly home after a circuitous outward trip. In doing so, they likely used stable visual room cues to return to their home base. When blindfolded and presented with a polygon track, the control rats still took a direct route home, ignoring the scent track and thus “closing the polygon.” In doing so they were likely integrating the self-movement cues generated on the outward trip.  In contrast to the ability of the control rats to switch from an spatial to a dead reckoning navigation strategy, the fimbria–fornix rats’ impairment in idiothetic navigation was starkly revealed when they wore blindfolds or were tested in infrared light. Rather than taking a direct route home after following a polygon track, they relocated the scent track and retraced their route back to the starting location. Consequently, their homebound route was as circuitous as their outbound route. This was not simply a matter of conveniently “taking an easy route home.” When the scent track was removed, the fimbria–fornix rats became disoriented, ate their food on the table, and then walked around almost haphazardly until they regained the refuge. Thus, in a situation in which they were forced to use a dead reckoning strategy, they were unable to do so. This result fails to confirm the suggestion that hippocampal rats preferentially use a dead reckoning navigation strategy (Pearce et al., 1998).

### Conclusion

Contemporary research suggests that the hippocampus plays some central role in spatial behavior [but see Squire (1992)], but there are divergent views concerning this role (O’Keefe and Nadel, 1978; Worden, 1992; Muller et al., 1996; Whishaw and Maaswinkel, 1998). The present finding supports the idea that the hippocampus plays a role in dead reckoning navigation. This conclusion is consistent with previous suggestions that the hippocampus contains an innate spatial framework within which it can generate vectors between points (Whishaw et al., 1995a,b, 1997; McNaughton et al., 1996; Samsonovich and McNaughton, 1997). Although the present results support a role for the hippocampus in dead reckoning navigation, they are not definitive in assigning this as an exclusive function. Hippocampal animals are also impaired in learning new spatial problems in which the demands of working memory are high (Shapiro and O’Connor, 1992; Angeli et al., 1993; Whishaw and Jarrard, 1995, 1996). This may mean that dead reckoning information aids in the transition between one problem and the next (Whishaw et al., 1997), or it may indicated that the impairment in dead reckoning navigation is but one aspect of a general hippocampal function in spatial behavior and memory (O’Keefe and Nadel, 1978).
